# Primary Cutaneous Nocardiosis in an Adolescent with Crohn Disease

**DOI:** 10.1155/2020/1532875

**Published:** 2020-11-10

**Authors:** Steven A. Svoboda, Joshua D. Eikenberg

**Affiliations:** ^1^Virginia Tech Carilion School of Medicine, Roanoke, VA, USA; ^2^Section of Dermatology, Virginia Tech Carilion School of Medicine, Roanoke, VA, USA

## Abstract

*Nocardia* is an aerobic, Gram-positive, partially acid-fast bacterium that often manifests as pulmonary infection since the primary route of entry is via the respiratory tract. As an opportunistic organism, *Nocardia* primarily affects immunocompromised individuals. Infection with *Nocardia* is uncommon. Primary cutaneous nocardiosis which is caused by percutaneous inoculation is even more rare. Here, we report a case of primary cutaneous nocardiosis in an adolescent with Crohn disease receiving treatment with adalimumab and azathioprine. Early identification and treatment are important to prevent disease progression and to avoid severe complications. Diagnosis is made principally by culture. Given that culture results may take up to two weeks to return, primary cutaneous nocardiosis should be maintained in the differential for any superficial cutaneous infection that arises in individuals undergoing treatment with immunosuppressive agents.

## 1. Introduction

The *Nocardia* species are aerobic, filamentous, Gram-positive, partially acid-fast bacteria that can cause localized or systemic disease. As an opportunistic organism, *Nocardia* most often affects immunocompromised individuals. Nocardiosis typically manifests as an isolated pulmonary infection following inhalation; however, disseminated disease may involve other organs including the brain and skin [1–3].

Although *Nocardia* infection is uncommon, primary cutaneous nocardiosis which is caused by direct skin inoculation is exceptionally rare [4–8]. Cutaneous infection may be characterized by nodules, ulcerations, pyoderma, cellulitis, or subcutaneous abscess formation [4]. Early diagnosis and treatment is important as infection can lead to life-threatening complications such as necrotizing nocardiosis [7]. Here, we report a case of primary cutaneous nocardiosis in an adolescent receiving immunosuppressive therapy for her Crohn disease (CD).

## 2. Case Presentation

A 13-year-old female with a history of CD presented to the dermatology clinic for a pruritic rash on the left upper back that started off as a “pimple” two weeks prior. The patient's medication regimen for the treatment of her CD included adalimumab 40 mg/0.4 ml once every other week and azathioprine 75 mg once daily. She had been on azathioprine for several years and adalimumab for approximately six months.

**Figure 1:**
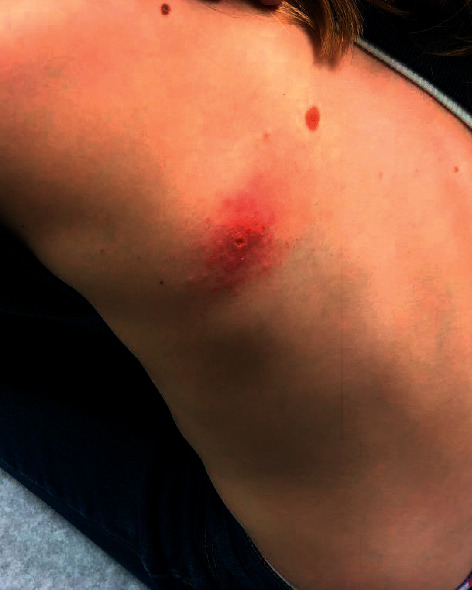
An edematous and erythematous plaque with purulent drainage on the left upper back.

Examination of the skin revealed an edematous and erythematous plaque with purulent drainage present on the left upper back (Figure 1). Empiric treatment with cephalexin was initiated while wound cultures were pending. Upon initial bacterial culture results returning positive for *Nocardia*, cephalexin was switched for minocycline. This antibiotic was selected due to the patient's previous adverse reactions to sulfonamide medications (rash) and to doxycycline (vomiting/diarrhea).

A punch biopsy was subsequently performed demonstrating pseudoepitheliomatous squamous hyperplasia, microabscesses, and granulomatous inflammation. This reaction pattern was consistent with an infectious etiology although no organisms were identified on Gram or Fite stain. Matrix-assisted laser desorption/ionization time-of-flight mass spectrometry was performed on the culture to identify the species as *Nocardia nova*, thus confirming the diagnosis of primary cutaneous nocardiosis. The patient was later transitioned to azithromycin based on the antibiotic susceptibility profile of the identified *Nocardia* species.

## 3. Discussion

In this case, primary cutaneous nocardiosis occurred in an adolescent with CD undergoing treatment with azathioprine and adalimumab, two immunosuppressive agents. While it is likely that immunosuppression played a role in the pathogenesis of this patient's infection, primary cutaneous nocardiosis has also been reported to occur in immunocompetent individuals [1, 3, 6]. The majority of cases of primary cutaneous nocardiosis present as a localized superficial infection often with a nodular or pustular appearance as seen in our patient. However, progression to cellulitis or abscess formation can occur [7]. Other manifestations may include lymphocutaneous involvement and mycetomas [8].

The diagnosis of cutaneous nocardiosis is primarily made by culture. As *Nocardia* are slow-growing organisms, culture results may take one to two weeks [7]. Although not as sensitive as culture, smear or touch preparation from skin biopsy specimens can be stained with Gram stain or acid-fast stain and facilitate rapid diagnosis within an hour [9, 10]. Interestingly, *N. nova* was isolated in our patient despite the most frequently isolated species being *N. brasiliensis*, followed by *N. asteroids* and *N. otitidiscaviarum* [6, 8]. Other diagnoses to maintain in the differential include superficial skin infections caused by *Staphylococcus aureus* and Streptococci species [7].

Treatment of primary cutaneous nocardiosis typically requires one to four months of antibiotic therapy [1, 2]. More severe cutaneous involvement may require prolonged treatment [2, 7]. Initiating appropriate antibiotics early on is important, especially in immunocompromised individuals, given the high mortality rate associated with these infections [2, 3]. Trimethoprim-sulfamethoxazole provides effective coverage against most *Nocardia* isolates [1, 8]. In the event of drug resistance or sulfonamide allergy, alternative medications may include cotrimoxazole, minocycline, amikacin, third-generation cephalosporins, ciprofloxacin, imipenem, and clindamycin [1, 8]. While empiric treatment should be initiated early on, antibiotic susceptibility testing should be performed due to the high rates of antibiotic resistance seen among the *Nocardia* species [1, 3, 4].

Primary cutaneous nocardiosis remains a diagnostic challenge and should be maintained in the differential for any superficial cutaneous infection that arises in immunocompromised individuals or in those receiving immunosuppressive treatment.
